# Association between left atrial low-voltage area and induction and recurrence of macroreentrant atrial tachycardia in pulmonary vein isolation for atrial fibrillation

**DOI:** 10.1007/s10840-024-01760-8

**Published:** 2024-02-06

**Authors:** Koichiro Sonoda, Tadatomo Fukushima, Asumi Takei, Kaishi Otsuka, Shiro Hata, Hiroki Shinboku, Takahiro Muroya, Koji Maemura

**Affiliations:** 1 Department of Cardiology, Sasebo City General Hospital, 9-3 Hirase-cho, Sasebo, 857-8511 Japan; 2 Department of Cardiovascular Medicine, Nagasaki University Graduate School of Biomedical Sciences, 1-7-1 Sakamoto, Nagasaki, 852-8501 Japan

**Keywords:** Atrial fibrillation ablation, Low-voltage area, Atrial tachycardia, Atrial remodeling, 3D mapping

## Abstract

**Background:**

The relationship between induction and recurrence due to atrial tachycardia (AT) and left atrial (LA) matrix progression after atrial fibrillation (AF) ablation remains unclear.

**Methods:**

One hundred fifty-two consecutive patients with paroxysmal and persistent AF who underwent pulmonary vein isolation (PVI) and cavo-tricuspid isthmus (CTI) ablation and achieved sinus rhythm before the procedure were classified into three groups according to the AT pattern induced after the procedure: group N (non-induced), F (focal pattern), and M (macroreentrant pattern) in 3D mapping.

**Results:**

The total rate of AT induction was 19.7% (30/152) in groups F (*n* = 13) and M (*n* = 17). Patients in group M were older than those in groups N and F, with higher CHADS_2_/CHA_2_DS_2_-VASc values, left atrial enlargement, and low-voltage area (LVA) size of LA. The receiver operating characteristic curve determined that the cut-off LVA for macroreentrant AT induction was 8.8 cm^2^ (area under the curve [AUC]: 0.86, 95% confidence interval [CI]: 0.75–0.97). The recurrence of AT at 36 months in group N was 4.1% (5/122), and at the second ablation, all patients had macroreentrant AT. Patients with AT recurrence in group N had a wide LVA at the first ablation, and the cut-off LVA for AT recurrence was 6.5 cm^2^ (AUC 0.94, 95%CI 0.88–0.99). Adjusted multivariate analysis showed that only LVA size was associated with the recurrence of macroreentrant AT (odds ratio 1.21, 95%CI 1.04–1.51).

**Conclusions:**

It is important to develop a therapeutic strategy based on the LVA size to suppress the recurrence of AT in these patients.

**Graphical abstract:**

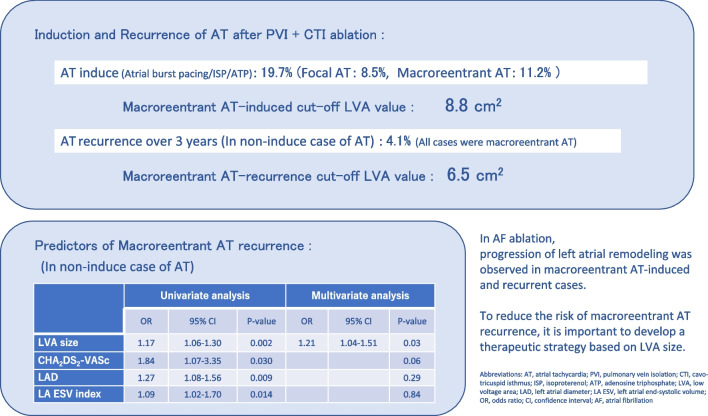

## Introduction

The Substrate and Trigger Ablation for Reduction of Atrial Fibrillation Trial Part II (Star AF II) study showed that procedures beyond catheter ablation (CA), other than pulmonary vein isolation (PVI), did not decrease the recurrence rate of atrial fibrillation (AF) in patients with persistent AF (PeAF) [1]. Additionally, patients with a larger left atrial low-voltage area (LVA) experienced a higher rate of AF recurrence [2, 3]. Treatment for LVA has been shown to improve the rate of sinus rhythm maintenance for patients with paroxysmal AF (PAF) [4, 5] or PeAF [6–8]. LVAs are associated with high inducibility of atrial tachyarrhythmias after PVI. In addition, the distribution of LVAs is specific for each type of macroreentrant atrial tachycardia (AT) [9]. This study aimed to investigate the relationship between induction and recurrence of AT and LVA after AF ablation while assessing potential predictive factors for AT recurrence.

## Methods

### Study design and patient population

**Fig. 1:**
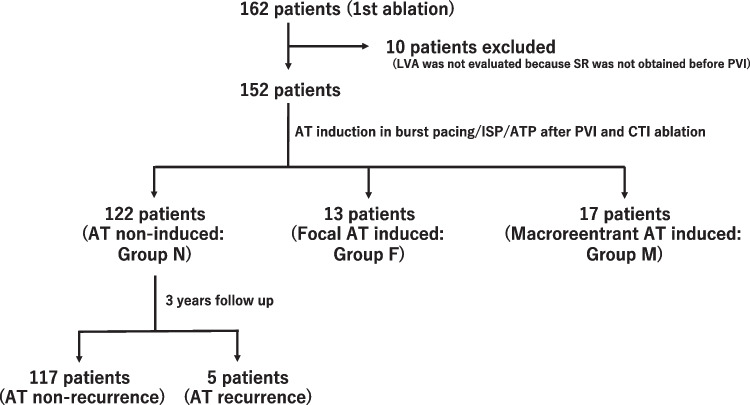
Of the 162 patients, 152 patients with sinus rhythm before mapping were included. Depending on the presence or absence of AT induction, they were classified as groups N, F, and M. Group N was divided into two groups based on the recurrence of AT and analyzed. N, non-induced atrial tachycardia; F, focal atrial tachycardia; M, macroreentrant atrial tachycardia; LVA, low-voltage area; SR, sinus rhythm; PVI, pulmonary vein isolation; ISP, isoproterenol; ATP, adenosine triphosphate, CTI ablation, cavo- tricuspid isthmus ablation; AT, atrial tachycardia

In total, 162 new consecutive cases of PAF and PeAF ablation were performed at our hospital from January 2015 to December 2016, with 152 cases (PAF, 81 cases; PeAF, 71 cases) achieving sinus rhythm before the voltage mapping and ablation procedure. In the case of PeAF, we included cases in which sinus rhythm was restored by internal cardioversion. After PVI and CTI ablation, we performed induction of supraventricular arrhythmia. In this study, CTI ablation was performed to exclude recurrence due to atrial flutter. None of the cases had a clinical history of AT. AT was defined as regularly cycled supraventricular tachycardia occurring after PVI and CTI ablation. Patients were divided into groups N (non-induced), F (focal pattern), and M (macroreentrant pattern), based on the presence or absence of provocation and the 3D pattern. We examined the patients’ background, left atrial LVA, and AT recurrence over 36 months (Fig. 1).

### Voltage mapping

Two 3D mapping systems were used: EnSite NavX (St. Jude Medical, Minneapolis, MN) and CARTO 3 (Biosense Webster, Diamond Bar, CA). Further, voltage mapping of the left atrium (LA) was conducted during sinus rhythm using a 20-pole circular mapping catheter (CMC), either Optima (St. Jude Medical) for EnSite NavX or LASSO (Biosense Webster) for CARTO 3.

In order to obtain as uniform a mapping as possible, the mapping catheter was firmly fixed to the geometric surface using a steerable sheath for the amount of time required to obtain a stable electrogram. The voltage amplitude of each acquired point was evaluated based on the peak-to-peak bipolar electrogram voltage using a bandpass filter set between 30 and 300 Hz. The LVA was defined as a voltage < 0.5 mV in sinus rhythm [10]. LVA was defined as a site with three or more adjacent low voltage (< 0.5 mV) points separated from each other by less than 5 mm. The area was measured using 3D mapping and was considered present when it occupied an area of 2 cm^2^ or more. For PeAF cases, internal cardioversion (15–30 J) using a BeeAT system (Japan Lifeline, Tokyo, Japan) was performed to restore sinus rhythm; however, 10 cases in which sinus rhythm could not be maintained by defibrillation were excluded from the study. The efficient internal cardioversion energy delivery enables the termination of atrial fibrillation at low energy without compromising efficacy. It also minimizes the risk of patients getting burned around the ground pad.

### Electrophysiology test and ablation procedure

The electrophysiology test and ablation procedure were performed under deep sedation with propofol, and an I-gel supraglottic airway device (Intersurgical, Wokingham, UK) was used for airway management. PVI and CTI ablation were carried out using a FlexAbility catheter (St. Jude Medical) with the EnSite or a SmartTouch or SmartTouch SF catheter (Biosense Webster) with the CARTO 3 by dragging at 30 W (25 W in the esophagus). The procedures were performed with each radiofrequency application limiting the temperature to 42 ℃ and overlapping with the 4 mm tag for 25–35 s. Following PVI and CTI ablation, induction of supraventricular arrhythmia was performed by administration of isoproterenol (ISP) 10 μg iv and then adenosine (ATP) 20 mg iv and burst pacing (300–180 ms: 15 beats, 190 ms: 30 beats) from the right atrium (RA). Induced arrhythmias with regular cycles were defined as AT and classified into groups N, F, and M based on the presence or absence of induction and 3D pattern. Group F (focal pattern) was defined as activation with a centrifugal pattern from a single focus, not recorded over the entire AT cycle length. Since it is difficult to differentiate between microreentry AT and focal AT, the focal AT pattern classification includes both types of AT in 3D mapping. Group M (macroreentrant pattern) was defined as activation recorded over the entire AT cycle length, circling around an anatomical or functional block line (large or local) characterized by a passage of slow conduction visually identified on the map. Sustained AT was analyzed using the activation map of 3D mapping, and non-sustained AT was induced with reproducibility and analyzed by 3D activation mapping using multiple electrodes as a guide. Induced AT was treated with additional therapy. Focal AT underwent focal ablation at the earliest excitation site. In case of multifocal AT, they were identified one by one, and as many areas as possible were treated. Regarding macroreentrant AT, in the case of the LA, we tried to ablate the slow conduction zone in 3D as much as possible, and if LVA was present around it, we decided to form a line (anterior line was the most). Additionally, when the anterior line was drawn, a roof line was added to prevent roof-dependent AT, and a bottom line was also added if necessary. If there was no LVA in the anterior wall, the mitral isthmus line was selected. In the case of the RA, the slow conduction region was ablated. The ablation procedure was terminated after confirming the elimination of AT inducibility and complete block line.

Non-pulmonary vein (PV) foci spontaneously induced reproducibly by administration of ISP and ATP, and cardioversion after AF induction by burst pacing, were identified by a self-reference mapping technique [11], and additional non-PV foci treatments were performed. The AF induced by burst pacing became sinus rhythm at internal cardioversion, and if no AF initiation occurred, it was followed up.

### Follow-up

Follow-up examinations were conducted at 1, 3, 12, 24, and 36 months after ablation, and Holter electrocardiography (ECG) was performed after 3 months. The regular physicians of each patient performed the intervening follow-ups. Recurrence of supraventricular arrhythmia was confirmed either by Holter ECG, an event recorder based on observations made through regular ECG, or from symptoms, and AF lasting more than 30 s was classified as a recurrence.

### Statistical analysis

Statistical analysis was performed using Rstudio, version 1.4.1717. Continuous variables are shown as mean ± standard deviation. For comparison, continuous variables were analyzed using the unpaired *t*-test, one-way analysis of variance, and the Kruskal–Wallis rank-sum test. Categorical variables were analyzed using the chi-squared test and logistic regression, and *P*-values < 0.05 were considered statistically significant. The receiver operating characteristic (ROC) curve was constructed by plotting sensitivity vs. specificity and used to distinguish the power of parameters in identifying the macroreentrant AT.

## Results

### Induction of atrial tachycardia

**Fig. 2:**
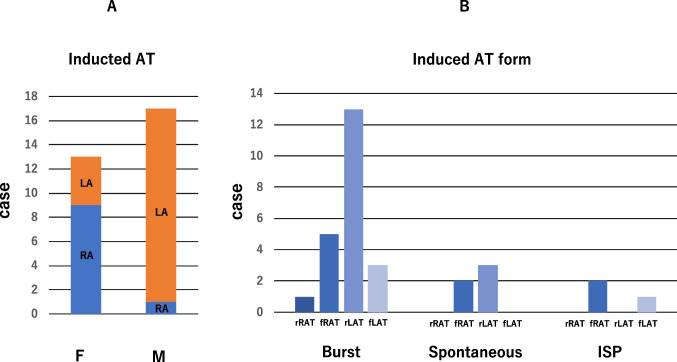
**A** AT was induced more in the right atrium in the focal type and in the left atrium in the macroreentrant type. **B** Burst pacing induced mostly left atrial reentrant tachycardia, and ISP stress was the only focal type. AT, atrial tachycardia; F, focal atrial tachycardia; M, macroreentrant atrial tachycardia; rRAT, reentrant right atrial tachycardia; fRAT, focal right atrial tachycardia; rLAT, reentrant left atrial tachycardia; fLAT, focal left atrial tachycardia; Burst, burst pacing; ISP, isoproterenol

AT was induced by the administration of ISP, RA burst pacing, and spontaneous onset. The overall AT induction rate after PVI and CTI ablation was 19.7% (30/152; Table 1). There were 13 cases in group F (8.5%) and 17 in group M (11.2%; Fig. 1). In group F, many cases were of right atrial origin (RA 9 cases/LA 4 cases), and in group M, most cases were of left atrial origin (RA 1 case/LA 16 cases; *P* = 0.0004; Fig. 2A), mitral isthmus–dependent AT in 15 cases (figure-of-eight AT in 5 cases), roof-dependent AT in one case, and RA crista AT in one case. Mitral isthmus–dependent AT corresponded to macroreentrant AT with slow conduction on the anterior wall in 3D mapping. Additionally, in group F, non-sustained AT was observed in 10 cases, sustained AT in three cases, and multifocal AT in two cases (all in the RA). In group M, all cases were sustained. Figure 2B shows the appearance of AT according to the induction procedure. Burst pacing tended to induce more macroreentrant AT in the LA, while ISP loading only induced focal AT.

**Table 1:** Induction of atrial tachycardia

	Atrium	Number of cases	Region
Focal AT	RA	9	Lateral 3, posterior 2, crista 2, multiple 2
	LA	4	Lateral 2, inferior 1, posterior 1
Macroreentrant AT	RA	1	Crista 1
	LA	16	Mitral 15 (figure-eight 5), roof 1

*AT* atrial tachycardia, *RA* right atrium, *LA* left atrium

### Induction of non-PV foci

Non-PV foci had either a spontaneous onset or were induced by the administration of ISP and ATP and cardioversion after AF induction by burst pacing. There were 8 cases of non-PV foci induced, all of them in group N. Non-PV foci were present at the superior vena cava (SVC) in four cases (spontaneously, after ISP, after ATP, and after cardioversion) and at the RA in four cases (spontaneously in two cases and after ATP in two cases).

### AF induction by burst pacing

AF was not induced in 104 out of 152 cases. Forty-eight cases were induced with AF (median burst pacing cycle 190 ms [240–180 ms], non-sustained in 30 cases, sustained in 18 cases). There was no difference in LVA size between non-induced and induced AF groups (4.5 ± 9.1 cm^2^ vs. 4.4 ± 8.1 cm^2^; *P* = 0.981). Induction of AF/AT resulted in a significantly wider LVA compared to non-induction (7.0 ± 12.4 cm^2^ vs. 3.1 ± 5.5 cm^2^; *P* = 0.008). Burst pacing was not performed after AT induction.

### Patients characteristics

#### Distribution of LVA in the LA

The proportions of LVA in the LA were anterior, 55.3%; posterior, 19.2%; septal, 16.1%; inferior, 7.4%; and roof, 2.0%. LA volume (left atrial end-systolic diameter index) and LVA were correlated (*R* = 0.29, *P* = 0.0006). A comparison of the LVA of PAF vs. PeAF showed that the LVA of PeAF was significantly wider (3.0 ± 6.7 cm^2^ vs. 6.1 ± 10.6 cm^2^; *P* = 0.03).

#### Characteristics among the three groups

**Fig. 3:**
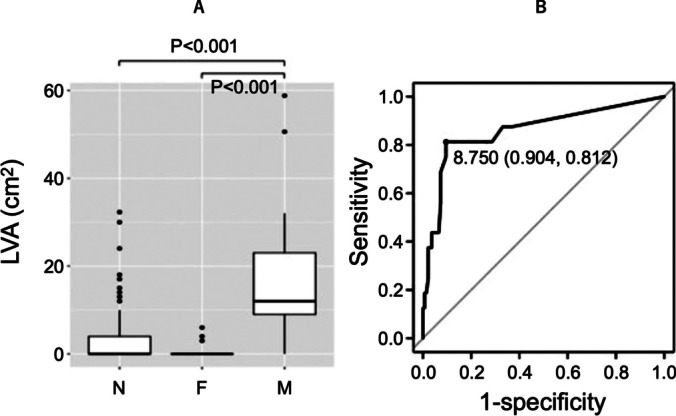
**A** The M group had significantly more LVA than the N/F groups (*P* < 0.001). **B** In the ROC curve, the macroreentrant AT-induced cut-off value was 8.8 cm^2^; specificity, 0.90; sensitivity, 0.81; AUC, 0.86 (95%CI: 0.75–0.97). LVA, low-voltage area; N, non-induced atrial tachycardia; F, focal atrial tachycardia; M, macroreentrant atrial tachycardia; ROC, receiver operating characteristic; AUC, area under the curve; CI, confidence interval

Table 2 shows the patient characteristics among the three groups (groups N, F, and M). There was no difference in the PAF/PeAF ratio and sex between the three groups. Patients in group M had a significantly higher age compared with those of groups N and F (group N vs. F vs. M, 64.5 ± 11.1 vs. 63.4 ± 11.5 vs. 76.1 ± 5.4; *P* < 0.001). Similarly, patients in group M were found to have higher CHADS_2_ (group N vs. F vs. M, 1.2 ± 1.2 vs. 1.1 ± 1.0 vs. 2.0 ± 1.2; *P* = 0.035), CHA_2_DS_2_-VASc (group N vs. F vs. M, 2.2 ± 1.6 vs. 2.2 ± 1.8 vs. 3.8 ± 1.5; *P* = 0.002), left atrial diameter (LAD) (group N vs. F vs. M, 40 ± 7 mm vs. 37 ± 8 mm vs. 43 ± 4 mm; *P* = 0.039), and left atrial end-systolic diameter (LA ESD) (group N vs. group F vs. group M, 73 ± 24 mL vs. 60 ± 15 mL vs. 84 ± 24 mL; *P* = 0.042). The LA ESD index (group N vs. F vs. M, 43 ± 14 mL/m^2^ vs. 36 ± 9 mL/m^2^ vs. 55 ± 19 mL/m^2^; *P* = 0.003) and LVA presence were significantly higher in group M (group N vs. F vs. M, 47 (38.5%) vs. 3 (23.1%) vs. 14 (82.4%); *P* = 0.001). In the comparison of LVA size (Fig. 3A), the LVA in group M was significantly wider (group N vs. F vs. M, 3.1 ± 5.8 cm^2^ vs. 1.0 ± 2.0 cm^2^ vs. 17.1 ± 16.8 cm^2^; *P* < 0.001). The LVA cut-off for macroreentrant AT induction according to the ROC analysis was 8.8 cm^2^ (area under the curve [AUC]: 0.86, 95% confidence interval [CI]: 0.75–0.97; Fig. 3B).

**Table 2:** Patient characteristics among the three groups (groups N, F, and M)

	N (*n* = 122)	F (*n* = 13)	*M* (*n* = 17)	*P*-value
Age (years)	64.5 ± 11.1	63.4 ± 11.5	76.1 ± 5.4	< 0.001
Sex: male	82 (67.2%)	10 (76.9%)	8 (47.1%)	0.176
BMI	23.8 ± 3.6	22.4 ± 4.0	23.4 ± 4.0	0.446
Type of atrial fibrillation: PAF	63 (51.6%)	7 (53.8%)	11 (64.7%)	0.599
Dialysis	5 (4.1%)	1 (7.7%)	0 (0%)	0.567
Basedow	4 (3.3%)	0 (0%)	0 (%)	0.611
Heart failure	21 (17.4%)	3 (23.1%)	4 (25.0%)	0.695
Hypertension	63 (52.1%)	6 (46.2%)	13 (81.2%)	0.072
DM	21 (17.4%)	4 (30.8%)	2 (12.5%)	0.407
Emboli	14 (11.6%)	0 (0%)	1 (5.9%)	0.353
CHADS_2_	1.2 ± 1.2	1.1 ± 1.0	2.0 ± 1.2	0.035
CHA_2_DS_2_-VASc	2.2 ± 1.6	2.2 ± 1.8	3.8 ± 1.5	0.002
AF duration (years)	1.4 ± 2.6	0.8 ± 0.7	1.1 ± 2.0	0.828
Arrhythmia on drug	9 (7.5%)	1 (7.7%)	3 (20.0%)	0.27
Betablocker	47 (38.5%)	7 (53.8%)	8 (47.1%)	0.484
EF (%)	60.0 ± 12.3	56.8 ± 11.3	65.0 ± 9.0	0.147
LAD (mm)	40.1 ± 6.7	36.8 ± 7.9	43.1 ± 4.0	0.039
LA ESV (ml)	73.5 ± 23.9	59.7 ± 14.6	83.8 ± 24.2	0.042
LA ESV index (ml/m^2^)	43.3 ± 13.8	36.4 ± 8.7	55.4 ± 18.7	0.003
AT induction				< 0.001
No induction	122 (100%)	0 (0%)	0 (0%)	
Burst pacing	0 (0%)	8 (61.5%)	14 (82.4%)	
Isoproterenol	0 (0%)	3 (23.1%)	0 (0%)	
Spontaneous	0 (0%)	2 (15.4%)	3 (17.6%)	
Low-voltage area case	47 (38.5%)	3 (23.1%)	14 (82.4%)	0.001
Low-voltage area (cm^2^)	3.1 ± 5.8	1.0 ± 2.0	17.1 ± 16.8	< 0.001
Anterior	1.8 ± 3.4	0.6 ± 1.1	8.2 ± 6.5	< 0.001
Posterior	0.6 ± 1.6	0.2 ± 0.8	3.1 ± 3.9	< 0.001
Inferior	0.3 ± 1.2	0	1.1 ± 2.2	0.017
Septal	0.4 ± 1.1	0.2 ± 0.5	3.2 ± 3.6	< 0.001
Roof	0.04 ± 0.3	0	0.5 ± 1.4	0.005
Lateral	0.03 ± 0.3	0	1.0 ± 2.9	0.001

Continuous variables are shown as the mean ± SD and categorical variables as frequency (%)

*BMI* body mass index, *PAF* paroxysmal atrial fibrillation, *DM* diabetes mellitus, *AF* atrial fibrillation, *EF* ejection fraction, *LAD* left atrial diameter, *LA ESV* left atrial end-systolic volume, *AT* atrial tachycardia

### Additional treatment in the three groups

#### Treatment of AT after induction

**Fig. 4:**
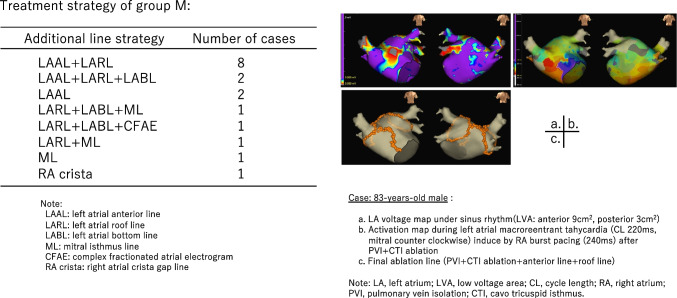
The treatment strategy and number of cases for group M are shown on the left side. One case is shown on the right side

There was no additional treatment in group N, while group F underwent focal ablation at the earliest excitation site. In cases of multifocal AT, as many sites as possible were treated. Figure 4 shows the treatment strategy and one case of group M. In group M, slow conduction zone (SCZ) of AT in the 3D activation map was observed in the LVA of the anterior wall in 13 out of the 17 cases. The line formation was as follows: left atrial anterior line (LAAL) + left atrial roof line (LARL) in eight cases, LAAL + LARL + left atrial bottom line (LABL) in two cases, LAAL in two cases, LARL + LABL + mitral isthmus line (ML) in one case, LARL + LABL + complex-fractionated atrial electrogram (CFAE) in one case, LARL + ML in one case, ML in one case, and right atrial crista gap line ablation in one case. Pacing was performed on the contralateral side near the line, and a 3D activation map confirmed the bidirectional conduction block.

A case is shown in Fig. 4 (right). In an 83-year-old male with PAF, left atrial voltage map during sinus rhythm revealed LVA measuring 9 cm^2^ in the anterior wall and 3 cm^2^ in the posterior wall. RA burst pacing (240 ms) after PVI + CTI ablation induced mitral AT with a cycle length of 220 ms. On 3D mapping, AT was performed by rotating the mitral valve counterclockwise, and SCZ was observed in the anterior wall. AT was eliminated during anterior line creation. The roof line was added, and bidirectional blocks were verified for each line.

#### Treatment of AF after induction

For treatment of non-PV foci, the SVC was isolated if they originated from it. For other sites, AF initiation sites were ablated. After ablating non-PV triggers, we performed the same method again and confirmed non-inducibility. AF induction by burst pacing and internal cardioversion (if persistent) was performed to restore sinus rhythm. Thereafter, no treatment was provided except in instances of spontaneous onset.

### Recurrence rate of atrial arrhythmias over 3 years

Of the 152 cases, recurrent atrial arrhythmias occurred in 24 cases (15.8%), including 13 cases of AF and 11 of AT. In 18 cases, a second CA was performed. There were no significant differences in LVA area at first ablation (6.8 ± 5.6 cm^2^ vs. 9.5 cm^2^; *P* = 0.66) and AT/AT + AF appearance ratio (2/6 cases vs. 7/12 cases; *P* = 0.62) between cases with and without PV reconnection.

### Recurrence rate of atrial arrhythmias among the three groups

Table 3 shows the recurrence rate of atrial arrhythmias among the three groups. Non-recurrence of atrial arrhythmias in groups N, F, and M occurred in 105 cases (86.1%), 11 cases (84.6%), and 12 cases (70.6%) at 36 months, respectively (*P* = 0.261). On the other hand, recurrence of AF was observed in 12 cases (9.8%), one case (7.7%), and none of the cases (0.0%) in groups N, F, and M, respectively, with no significant difference among the three groups (*P* = 0.395). No AF recurrence was observed in patients treated with non-PV foci. The recurrence rate of AF with or without AF induction in burst pacing was 8/97 cases (8.2%) in the no-AF induction group and 4/55 cases (7.3%) in the AF induction group, with no significant difference (*P* > 0.99). Recurrence of AT occurred in five cases (4.1%), one case (7.7%), and five cases (29.4%) in groups N, F, and M, respectively, and was more common in group M (*P* = 0.001). Of the 11 cases with AT recurrence, 10 cases underwent a second CA, which was performed in all five cases of recurrent AT in group N. Persistent AT had a macroreentrant pattern associated with the left atrial LVA. One case of AT recurrence in group F was multifocal, and the second CA was a recurrence of right atrial focal AT. Of the five cases with AT recurrence in group M, four underwent a second CA, three had left atrial line gap recurrence, and one had left atrial focal AT unrelated to the LVA. In each case, line gap ablation and new focal AT ablation were performed. In one case, electrophysiology test and CA were not performed, and the patient was prescribed medication during the follow-up.

**Table 3:** Recurrence rate of atrial arrhythmias among the three groups

	N (*n* = 122)	F (*n* = 13)	M (*n* = 17)	*P*-value
AF/AT recurrence free	105 (86.1%)	11 (84.6%)	12 (70.6%)	0.261
AF recurrence	12 (9.8%)	1 (7.7%)	0 (0.0%)	0.395
2nd ablation	8	1	0	
	4 (PV gap), 4 (non-PV)	1 (non-PV)		
AT recurrence	5 (4.1%)	1 (7.7%)	5 (29.4%)	0.001
2nd ablation	5	1	4	
	5 (rLAT)	1 (fRAT)	3 (line gap), 1 (fLAT))	

*AF/AT* atrial fibrillation, *PV* pulmonary vein, *rLAT* reentrant left atrial tachycardia, *fRAT* focal right atrial tachycardia, *fLAT* focal left atrial tachycardia

### AT recurrence in non-induced cases of AT (group N, 122 cases)

#### Comparison between the non-AT and AT recurrence groups

During the 3-year follow-up in group N, 117 cases of non-AT recurrence and five cases of AT recurrence were found (Table 4). Second CA was performed in the AT-recurrent cases, and left reentrant AT was confirmed by electrophysiological testing in all five cases. Afterward, AT was eliminated through the treatment of SCZ. When compared with AT non-recurrence, patients with AT recurrence had a significantly higher age (64.1 ± 11.2 years vs. 74.2 ± 5.1 years; *P* = 0.046), CHA_2_DS_2_-VASc (2.1 ± 1.6 vs. 3.8 ± 1.9; *P* = 0.021), LAD (40 ± 7 mm vs. 49 ± 4 mm; *P* = 0.003), and LA ESD index (43 ± 13 mL/m^2^ vs. 61 ± 16 mL/m^2^; *P* = 0.007) at the first ablation.

**Table 4:** Comparison of the non-AT and AT recurrence groups in group N

	AT − (*n* = 117)	AT + (*n* = 5)	*P*-value
Age (years)	64.1 ± 11.2	74.2 ± 5.1	0.046
Sex: male	80 (68.4%)	2 (40.0%)	0.402
BMI	23.8 ± 3.7	23.7 ± 2.7	0.979
Type of atrial fibrillation: PAF	61 (52.1%)	2 (40.0%)	0.94
Dialysis	4 (3.4%)	1 (20.0%)	0.501
Basedow	4 (3.4%)	0 (0%)	1
Heart failure	19 (16.4%)	2 (40.0%)	0.446
Hypertension	60 (51.7%)	3 (60.0%)	1
DM	20 (17.2%)	1 (20.0%)	1
Emboli	13 (11.2%)	1 (20.0%)	1
CHADS_2_	1.2 ± 1.1	2.2 ± 1.8	0.057
CHA_2_DS_2_-VASc	2.1 ± 1.6	3.8 ± 1.9	0.021
AF duration (years)	1.5 ± 2.6	0.2 ± 0.1	0.415
Arrhythmia on drug	9 (7.8%)	0 (%)	1
Betablocker	45 (38.5%)	2 (40.0%)	1
EF (%)	59.5 ± 12.4	70.4 ± 5.2	0.054
LAD (mm)	39.8 ± 6.5	48.8 ± 4.3	0.003
LA ESV (ml)	72.8 ± 24.1	90.5 ± 7.2	0.147
LA ESV index (ml/m^2^)	42.6 ± 13.3	61.3 ± 15.7	0.007
Low-voltage area case	42 (35.9%)	5 (100%)	0.016
Low-voltage area (cm^2^)	2.6 ± 5.0	14.3 ± 10.4	< 0.001
Anterior	1.5 ± 2.9	8.5 ± 5.7	< 0.001
Posterior	0.6 ± 1.6	1.5 ± 2.1	0.22
Inferior	0.2 ± 1.1	1.0 ± 1.7	0.136
Septal	0.3 ± 0.9	2.3 ± 2.4	< 0.001
Roof	0.03 ± 0.3	0.4 ± 0.9	0.011
Lateral	0	0.6 ± 1.4	< 0.001

Continuous variables are shown as the mean ± SD and categorical variables as frequency (%)

*BMI* body mass index, *PAF* paroxysmal atrial fibrillation, *DM* diabetes mellitus, *AF* atrial fibrillation, *EF* ejection fraction, *LAD* left atrial diameter, *LA ESV* left atrial end-systolic volume, *AT* atrial tachycardia

#### Relationship between AT recurrence and LVA

**Fig. 5:**
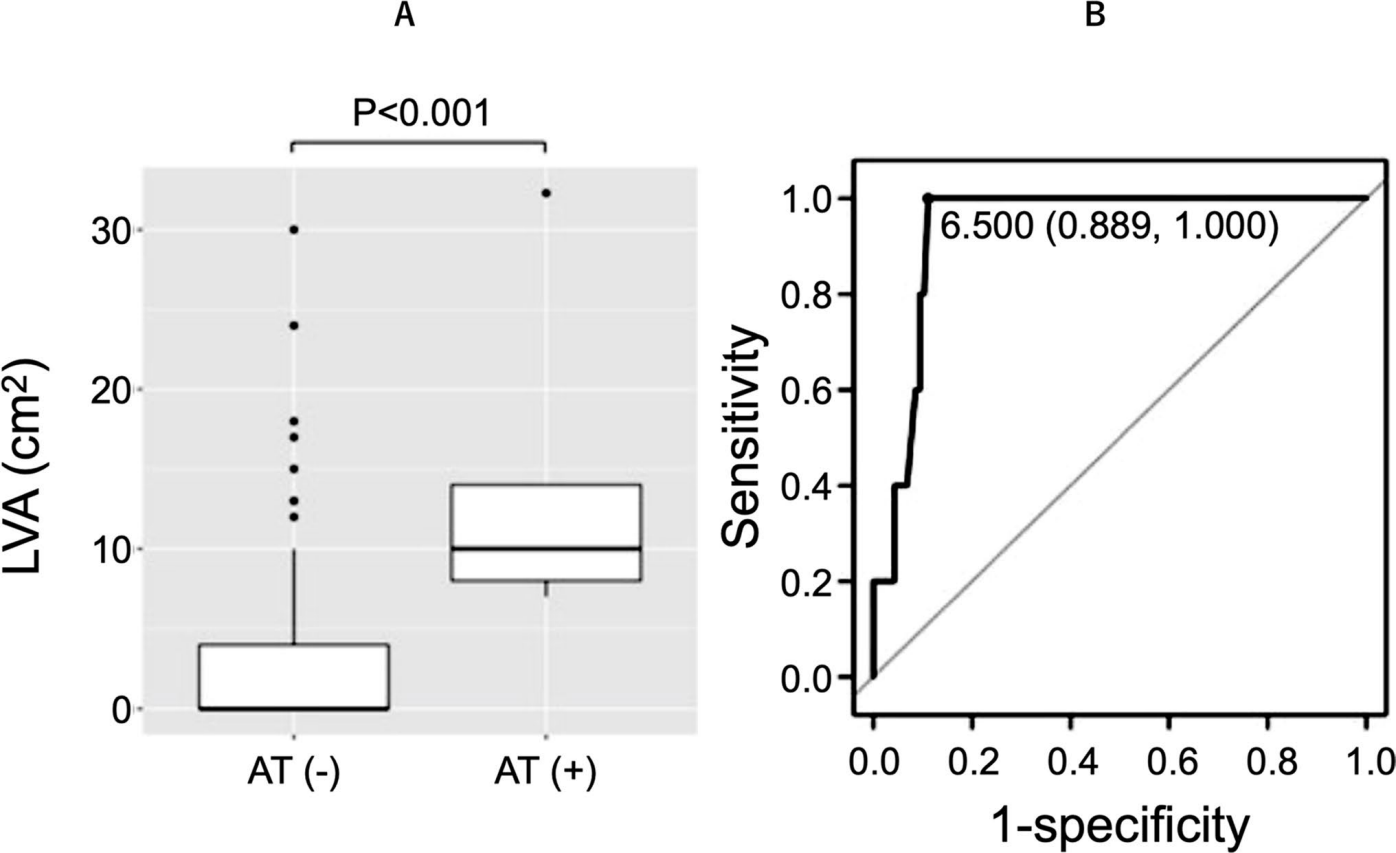
**A** The group with recurrent AT had significantly more LVA at the first ablation than the group without recurrent AT (*P* < 0.001). **B** In the ROC curve, the cut-off value of the LVA area at first ablation when macroreentrant AT recurred was 6.5 cm^2^, with a specificity of 1.00 and a sensitivity of 0.90, AUC 0.94 (95%CI: 0.88–0.99). LVA, low-voltage areas; AT( −), no recurrence due to atrial tachycardia, AT( +), recurrence due to atrial tachycardia; ROC, receiver operating characteristic; AUC, area under the curve; CI, confidence interval

In AT-recurrent cases, LVA size at the first ablation (2.6 ± 5.0 cm^2^ vs. 14.3 ± 10.4 cm^2^; *P* < 0.001) was high (Fig. 5A). The cut-off LVA at the time of the first ablation for AT recurrence was 6.5 cm^2^ (AUC 0.94, 95%CI 0.88–0.99; Fig. 5B). No correlation was found between age and LVA size (*P* = 0.07). Multivariate analysis adjusted for LVA size, CHA_2_DS_2_-VASc, LAD, and LA ESV index revealed that only LVA size was associated with AT recurrence (odds ratio 1.21, 95%CI 1.04–1.51; *P* = 0.03; Table 5).

**Table 5:** Univariate and multivariate analysis of predictors of AT recurrence in group N

	Univariate analysis			Multivariate analysis		
	OR	95%CI	*P*-value	OR	95%CI	*P*-value
LVA size	1.17	1.06–1.30	0.002	1.21	1.04–1.51	0.03
CHA_2_DS_2_-VASc	1.84	1.07–3.35	0.03			0.06
LAD	1.27	1.08–1.56	0.009			0.29
LA ESV index	1.09	1.02–1.7	0.014			0.84

*LVA* low-voltage area, *LAD* left atrial diameter, *LA ESV* left atrial end-systolic volume, *OR* odds ratio, *CI* confidence interval

### Relationship between recurrent AF and LVA

There were 13 cases of AF recurrence among all 152 cases. There was no significant difference in LVA between the group without AF recurrence (139 cases) and the group with AF recurrence (13 cases): 4.3 ± 8.9 cm^2^ vs. 5.8 ± 8.0 cm^2^ (*P* = 0.585).

## Discussion

Previous studies have found that the main cause of post-ablation AF recurrence is PV reconduction [12]. However, with the recent technological advances in areas such as contact force, the rate of PV reconduction is decreasing [13]. While additional procedures beyond PVI are needed to improve sinus rhythm maintenance, their effectiveness is reduced by the presence of LVA [2]. The electrical and structural substrate for AF is believed to result from a reduction in the refractory period and increased non-uniform anisotropy. Increased non-uniform anisotropy may be caused by changes in connexins or remodeling of atrial structures, such as dissociation of atrial bundles or endo- and perimysial fibrosis [14]. In patients with chronic AF, the severity of interstitial fibrosis between individual myocytes (endomysial) and atrial bundles (perimysial) has been shown to increase [15]. Fibroblasts are also known to induce ectopic activity and have been identified as arrhythmogenic [16]. Although atrial fibrosis has been suggested as the mechanism underlying low voltage [17], a direct connection between LVAs and histological fibrosis has yet to be confirmed. LVAs, which may consist of arrhythmogenic tissue, have been effectively treated by LVA ablation [4–8]. However, Yamaguchi et al. [18] found that LVA ablation in the LA did not significantly improve the maintenance of sinus rhythm and that the larger the LVA, the more difficult it is to maintain sinus rhythm. In addition, LVA includes conduction delay and blockage [10] and is considered an arrhythmic substrate for reentrant AT. It has been reported that 4–31% of macroreentrant AT appears after PVI [19, 20], and LVA ablation should be considered to reduce the recurrence rate.

### Relationship between LVA ablation and AT

Yang G et al. [6] divided patients with PeAF into two groups based on the presence or absence of LVA: a group without substrate and a group with substrate, which underwent additional ablation. The study found that AT recurrence was lower in the absence of substrate and increased over time despite substrate modification. Additionally, stepwise ablation with linear ablation and complex-fractionated electrograms has been reported to increase recurrence due to AT [6]. Yang et al. [21] conducted a randomized clinical trial in which 229 symptomatic non-PAF patients were randomized 1:1 to a STABLE-SR group (*n* = 114) or a conventional STEPWISE group (*n* = 115). After 18 months, 74.0% of the STABLE-SR group and 71.5% of the STEPWISE group were supraventricular arrhythmia-free, showing no significant difference (hazard ratio 0.78, 95%CI 0.47–1.29; *P* = 0.325). In addition, no significant difference in AT recurrence was identified. The only significant differences were in operating time, fluoroscopy time, and energy delivery time, which were all shorter in the STABLE-SR group. A meta-analysis of LVA ablation [22] consisting of six studies with a mean follow-up of 17 months found that LVA ablation, in addition to PVI, was more effective in preventing atrial arrhythmia recurrence than PVI alone or PVI with conventional wide empirical ablation. In particular, LVA ablation decreased the post-ablation AT recurrence compared with PVI plus conventional wide empirical ablation (14% vs. 46%; odds ratio 0.16, 95%CI 0.07–0.37). Furthermore, in the VOLCANO Trial [23], the AF/AT recurrence rate was similar in the LVA-treated group (B) and the untreated group (C), and the onset of AT increased in the B group (group B, 71% vs. C, 32%; *P* < 0.0001). This is because LVA ablation near an anatomical obstruction, such as the annulus, may unintentionally create an iatrogenic slow-conducting isthmus. Moreover, linear ablation to isolate the LVA may be inappropriate as it could create a conduction gap for a perfectly linear lesion. Further, new AT, such as biatrial tachycardia, appeared in the group that underwent LVA, suggesting an increase in iatrogenic AT.

In our study, macroreentrant pattern AT was observed in older patients with advanced left atrial remodeling and a wider left atrial LVA. On the other hand, focal pattern AT did not increase the LVA compared to the AT non-induced group. In the treatment of macroreentrant pattern AT in group M, the anterior LVA was a slow conduction zone in many cases as shown by 3D mapping, and an anterior line with slow conduction was often created. All patients with AT recurrence had LVA at the time of the first ablation, and 75% of those who underwent the second ablation had recurrence due to the gap in the anterior wall line. In this study, 94% of the patients in group M were treated using a catheter without contact focus. The use of lesion size index [24] and ablation index [25] may reduce the chronic phase line gap. Furthermore, it has been reported that transmural lesions and fewer AF recurrences are more likely to be obtained with high power-short duration ablation than with conventional ablation in the atrium [26–28]. High power-short duration ablation is considered to be done in LVA, linear ablation, and PVI.

### Regarding cases in which AT is not induced with LVA

It has been reported that PVI patients who had inducibility of any left atrial tachyarrhythmias and those with low-voltage areas during the initial procedure demonstrated lower recurrence-free survival rates [9]. In our study, even in the N group in which AT was not induced, 4.1% of patients had a recurrence of AT, many of which occurred in older patients with progressed left atrial remodeling. According to the ROC analysis, the cut-off LVA for AT induction during the first ablation was 8.8 cm^2^ and that for AT recurrence in the chronic phase was 6.5 cm^2^, even if AT was not induced. This may suggest that, as LVA progresses over time, AT becomes more likely to emerge. In the multivariate analysis, only the LVA size was associated with AT recurrence, not age or LA volume.

For ablation cases in older patients, it is important to avoid multiple ablations to suppress AT recurrence and medical costs. Therefore, even if AT is not induced, it is suggested that a therapeutic strategy (line formation including LVA) to suppress AT recurrence is important if the left atrial LVA is 6.5 cm^2^ or more.

Regarding AF recurrence, there was no difference in the recurrence rate of AF, regardless of whether AF was induced by burst pacing or not. This is because high-rate burst pacing was performed, and therefore, it is possible that non-clinical AF might have been induced. No association was found between the presence or absence of AF recurrence and LVA size (*P* = 0.585). In this study, AF recurrence was not observed in group M; however, recurrences of frequent atrial premature complexes or AF may have potentially transitioned to sustained AT.

### Limitations

In this study, two types of 3D-mapping CMCs (EnSite NavX and CARTO3) were used; nonetheless, it was reported that there was no difference in the potential acquisition [29]. Recently, the low-voltage area can now be evaluated more precisely using a recently developed electrode [30]; hence, it is deemed necessary to reexamine it with this new system, since the CMC may overestimate LVA. We used an induction method within a limited time after PVI, but if AT induction is additionally performed by programmed stimulation in the left atrium/from the left atrium, there may be a difference in the AT induction rate. Since it is difficult to differentiate between focal AT and microreentrant AT in 3D mapping, both types were included in the focal pattern. Furthermore, a wide LVA does not necessarily induce AT, and factors other than LVA (tissue conductance, among others) may also be involved in the induction of AT, but these were not investigated in this study.

## Conclusion

In AF ablation, progression of left atrial remodeling was observed in macroreentrant AT-induced and macroreentrant AT-recurrent cases. It is important to develop a therapeutic strategy based on LVA size to reduce the risk of macroreentrant AT recurrence.

## Data Availability

The datasets generated and/or analyzed during the current study are available from the corresponding author upon reasonable request.

## Declarations

### Permission to reproduce material from other sources

Not applicable .
